# Drug Resistance Modulation in *Staphylococcus Aureus*, a New Biological Activity for Mesoionic Hydrochloride Compounds

**DOI:** 10.3390/molecules16032023

**Published:** 2011-02-28

**Authors:** Cledualdo Soares de Oliveira, Vivyanne dos Santos Falcão-Silva, José Pinto Siqueira-Júnior, David Peter Harding, Bruno Freitas Lira, Jorge Gonçalo Fernandes Lorenzo, José Maria Barbosa-Filho, Petrônio Filgueiras de Athayde-Filho

**Affiliations:** 1 Department of Chemistry, Federal University of Paraíba, 58059, João Pessoa, PB, Brazil; E-Mails: aldoscarchi@yahoo.com.br (C.S.D.O.); kauaii@yahoo.com (D.P.H.); brunofrlira@hotmail.com (B.F.L.); 2 Laboratory of Pharmaceutical Technology, Federal University of Paraíba, 58051, João Pessoa, PB, Brazil; E-Mails: jgflorenzo@hotmail.com (J.G.F.L.); jbarbosa@ltf.ufpb.br (J.M.B.-F.); 3 Department of Molecular Biology, Federal University of Paraíba, 58059, João Pessoa, PB, Brazil; E-Mails: jpsiq@uol.com.br (J.P.S.-J.); vivyannefalcao@yahoo.com.br (V.D.S.F.-S.)

**Keywords:** mesoionic compounds, modulation of drug resistance, efflux pump inhibitor

## Abstract

Two salts of the mesoionic compounds 1,4-diphenyl-5-(5-nitro-2-furanyl)-1,3,4-thiadiazolium-2-thiol chloride (**MC-1**) and 4-phenyl-5-(5-nitro-2-furanyl)-1,3,4-thiadiazolium-2-phenylamine chloride (**MC-2**) were synthesized utilizing 1,4-diphenyl-thiosemicarbazide and 5-nitro-2-furoyl chloride as starting materials. Their structures were characterized by IR, ^1^H-NMR, ^13^C-NMR and elemental analysis. These compounds were analyzed for their influence on the effectiveness of norfloxacin, tetracycline, and erythromycin (standard antibiotics) against resistant strains of *Staphylococcus aureus.* **MC-1** and **MC-2**, at sub-inhibitory concentrations of 16 μg/mL, favourably modulated the antibiotic activity of tetracycline by 16- and 32-fold, respectively (MIC), and that of erythromycin by 4-fold.

## 1. Introduction

During the last few years, mesoionic compounds (MCs) have attracted the attention of chemists because of the bonding aspects of their unusual structures, being mesomeric heterocyclic betaines, strongly stabilized by π electron delocalization, and large dipole moments [1]. Due to their inherent structural characteristics, mesoionic compounds have aroused research interest in photonics, nonlinear optical studies [2,3,4,5,6,7,8] and medicinal chemistry, where a wide range of biological activities have been discovered, as summarized in several review articles [9,10,11]. They can be biologically active, for example, mesoionic 2-(4-chlorophenyl)-3-methyl-4-(4-methoxyphenyl)-1,3-thiazolium-5-thiolate induces vasorelaxation [12].

Biological studies have shown that hydrochlorides of mesoionic 1,3,4-thiadiazolium-2-thiolate compounds are active against Gram-positive and Gram-negative bacteria. Pharmacological effects at the cellular level (involving relaxation in smooth muscle tissue due to a confirmed locking mechanism for calcium channel blockers), were observed for 1,3-diphenyl-5-(5-nitro-2-furanyl)-1,3,4-triazolium-2-thiol chloride [13,14]. This locking mechanism is of great interest because it might represent a tool against multi-drug efflux pumps [15,16].

The resistance acquired by *Staphylococcus aureus* to virtually all antibiotics currently in use is of considerable concern. Resistant strains like IS-58, which has specific efflux proteins (TetK) that reduce the intracellular concentrations of tetracycline, have caused thousands of deaths, largely through bloodstream infections. Efflux pumps are known to be the main mechanism of bacterial resistance. Their function is to decrease intracellular drug concentration by return “pumping” the drug(s) to the extra cellular medium, allowing the bacteria to survive [17,18].

Efflux pumps are integral membrane proteins of bacteria that extrude antibiotics and other antimicrobial agents from the cell. They can be specific to a given compound or class of compounds or capable of removing a variety of structurally unrelated antimicrobial compounds. Resistance modification is the desired effect (to overcome or modulate bacterial resistance by reducing efflux pump activity). Efflux inhibition can restore the antibiotic’s activity against the bacteria throughco-administration [16,17].

In this study we describe the novel structure of a mesoionic compound hydrochloride **MC-2**. The results presented here represent the first report of derivatives of this class (heterocyclic betaines) as putative efflux pump modulators. The present findings indicate that 1,4-diphenyl-5-(5-nitro-2-furanyl)-1,3,4-triazolium-2-thiol chloride (**MC-1**), and 4-phenyl-5-(5-nitro-2-furanyl)-1,3,4-thiadiazolium-2-phenylamine chloride (**MC-2**), could serve as a source of heterocyclic compounds that modulate bacterial resistance, when used as adjuvants of antibiotics.

## 2. Results and Discussion

**Scheme 1:**
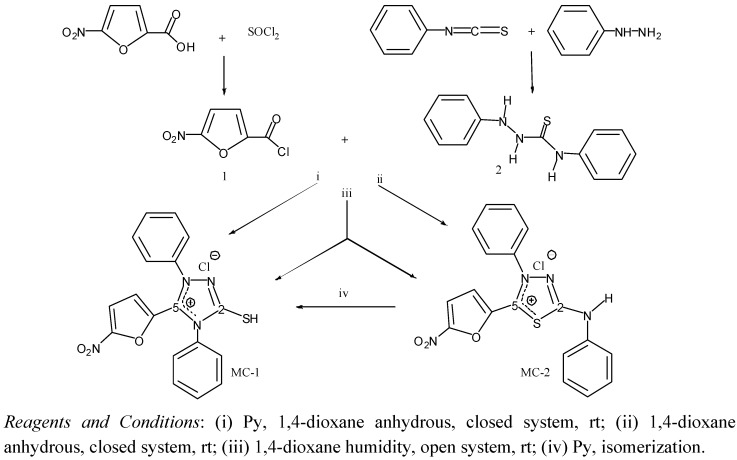
Procedure to obtain **MC-1** and **MC-2** from the chloride 5-nitro-2-furoyl and 1.4-diphenylthiosemicarbazide.

The merits of this research are in the discovery of two hydrochlorides representative of the mesoionic heterocycles class capable of inhibiting the bacterial resistance of *Staphylococcusaureus* against tetracycline and erythromycin. The compound **MC-2** is a novel structure and was obtained by the reaction of 5-nitro-2-furoyl chloride with 1,4-diphenylthiosemicarbazide. However, worth mentioning is that from this same reaction we also obtained the isomer **MC-1**, which was first synthesized by Athayde-Filho *et al.* in 1996 [14]. Although one synthesis is used for both compounds–1,3,4-triazolium-2-thiol and 1,3,4-thiadiazolium-2-phenylamine–each desired compound is easily obtained alone under controlled conditions. Thus, **MC-1** is a thermodynamic product obtained under anhydrous conditions in a closed system and in the presence of pyridine (route i, Scheme 1) while the kinetic product**MC-2** is obtained under anhydrous conditions in a closed system (route ii, Scheme 1). Under relatively humid conditions in an open system (route iii, Scheme 1), a mixture of isomers can be obtained and separated by column chromatography. **MC-1**can also being obtained from **MC-2** via alkaline isomerization (route iv, Scheme 1) [19,20].

**MC-2** is easily differentiated from its isomer **MC-1** by the usual physical methods of organic compound characterization. Absorption bands around 3,400 cm^−1^ with deformations related to the NH side chain appear in the infrared spectra of **MC-2**, which are confirmed by ^1^H-NMR *via* a singlet at around 13.00 ppm. In fact, although ^1^H-NMR studies are not very informative when there are no hydrogens in the heterocyclic ring, as in this case, they are however important for the detection of hydrogens in substituent groups. In contrast ^13^C-NMR spectroscopy is an important tool for characterizing structures of mesoionic compounds and their derivatives due to the large differences in electronic densities between the two regions (HOMO and LUMO) of the heterocyclic ring [21,22]. This difference of electronic density manifests itself through positive and negative shielding effects for the carbons of the heterocyclic ring and of the carbons of aromatic or aliphatic groups linked to the mesoionic ring. The C2 and C5 carbons have chemical shifts that distinguish the mesoionic isomers. For 1,3,4-triazolium-2-thiol type compounds (*i.e.*, **MC-1**), the chemical shifts of C2 and C5 appear around 169.8 and 148.2, respectively, while for the 1,3,4-thiadiazolium-5-phenylamine type compounds (*i.e.*, **MC-2**) the chemical shifts of C2 and C5 are centered around 160 and 165 ppm [19,20]. These results are consistent with the assignments of chemical shifts for **MC-1** and corroborate the assignments of carbons made for **MC-2**[14,23].

**MC-2** is an air stable solid, whose molecular formula was confirmed by spectroscopic data(^1^H-, ^13^C-NMR and infrared spectroscopy) which was in complete agreement with the proposed structures. The main absorption bands in the infrared spectrum: 3,444 (*ν*_N–H_); 1,620 (*ν*_C=C_); 1,574 (*ν*_C=N_); 1,538 and 1,345 cm^−1^ (*ν*_ΝO2 symmetric and asymmetric_). The ^1^H-NMR spectrum of **MC-2** shows twelve aromatic hydrogen signals between 7.93 and 6.67 ppm, while doublets integrating for two hydrogens each at 7.44 and 6.73 ppm with a coupling constant of 2.8 Hz are assigned to the furan ring and a singlet at 13.20 ppm to the phenylalanine NH hydrogen.

The ^13^C-NMR study of **MC-2** showed fourteen chemical shifts. Of these, six were assigned to the quaternary carbons and eight to the tertiary aromatic carbons. The carbons of the **MC-2** mesoionic ring have chemical shifts at 166.0 and 160.5 ppm, respectively, assigned to C2, C5. The benzene aromatic carbons have chemical shifts of 138.1, 137.6, 132.5, 130.5, 129.5, 125.7, 124.4 and 120.7 ppm, and the carbons of the furan ring have chemical shifts of 106.3, 118.5, 139.3 and 150.9 ppm; the last carbon is in a low field due to deshielding caused by the nitro group.

The synthesis and study of these mesoionic compound derivatives MC-1 and MC-2 expanded and we obtained the following data regarding bacterial resistance through *in vitro* microbiological assays: the compounds **MC-1** and **MC-2** alone showed relevant antibacterial activity against *Staphylococcus aureus*. Comparative studies of the MIC of tetracycline, erythromycin, and norfloxacin, with **MC-1** and **MC-2**, show that both heterocyclic compounds are active (MIC 64) against IS-58, less potent than tetracycline (MIC 32), however, against RN-4220-1, **MC-1** was twice as active as **MC-2**, yet both are substantially more active than erythromycin (MIC 256); neither showed greater activity againstSA-1199B than norfloxacin (Table 1).

**Table 1:** MIC values (µg/mL) of test strains for antibiotics and hydrochloride of mesoionic compounds.

Strain (efflux protein)	Tetracycline	Erythromycin	Norfloxacin	MC-1	MC-2
IS-58 (TetK)	32	-	-	64	64
RN-4220 (MrsA)	-	256	-	32	64
SA-1199B (NorA)	-	-	64	64	64

However, the second group of assays for bacterial resistance showed an unexpected result. Hydrochlorides of mesoionic compounds **MC-1** and **MC-2** enhanced the activity of tetracycline and erythromycin by greatly reducing the concentration necessary to inhibit the growth of drug resistant (effluxing) strains. When the **MC-1** or **MC-2** compounds were incorporated into the growth medium at 16 µg/mL (¼ MIC), a four-fold reduction in the MIC was observed for erythromycin, and for tetracycline the MIC was reduced by 16 and 32-fold when promoted by **MC-1** and **MC-2**, respectively (Table 2). No reduction in MIC was observed for norfloxacin, indicating that the mesoionic compounds do not act as inhibitors of the NorA efflux protein. All experiments were carried out in duplicate with consistent results.

**Table 2:** MIC values (µg/mL) of test strains for antibiotics in the absence and presence of **MC-1** and **MC-2** (16 µg/mL).

Strain /(efflux protein)/Antibiotic (MIC µg/mL)	Antibiotic MIC µg/mL with (*)MIC reduction factor in the presence of MC-1/MC-2(16 µg/mL)	
	Antibiotic + MC-1	Antibiotic + MC-2
IS-58/(TetK)/Tetracycline (32)	2 (16 *)	1 (32 *)
RN-4220/(MrsA)/ Erythromycin (256)	64 (4 *)	64 (4 *)
SA-1199B/(NorA)/Norfloxacin (64)	64 (0 *)	64 (0 *)

(X*)—MIC reduction factor.

Mesoionic compounds are planar five-membered ring betaines, which contain distinct regions of positive and negative charges associated with any poly-heteroatomic system, yet have a net neutral electrical character. These properties enable them to cross cellular membranes and interact strongly with biomolecules [24]. Hydrochlorides of the mesoionic compounds **MC-1** and **MC-2**effectively enhanced the antibiotic activity of tetracycline and erythromycin by reducing the concentrations needed to inhibit the growth of drug resistant (effluxing) strains. A lipophilic character, affecting membrane solubility, while common among efflux pump inhibitors, is likely to influence efflux transporter binding [18], and partial polarity interactions with the efflux transporter amino acid backbones, could combined to produce these results, however both potency modulation and antibacterial activity may be related to the nitro compounds present [25].

Besides being lipophilic, salts of mesoionic compounds have also been shown to block ion channels, see **MC-1**, a potent calcium and potassium channel blocker [14]. Markham and Neyfach [26] observed tetracycline-specific transporters and show that *B. subtilis* TetL and *S. aureus* Tetk, transport monovalent cations Na^+^ and K^+^ (genetic knockout of these transporters causes an increase in tetracycline sensitivity, and reduction in high salinity media growth). Further assessment of the biological mechanisms involved would be valuable seeing that calcium channel blockers are able to inhibit multidrug transporters or P-glycoprotein (Pgp) [27].

## 3. Experimental

### 3.1. General

Infrared spectra were obtained by means of a IFS66 Bruker spectrometer with the samples in KBr discs. ^1^H- and ^13^C-NMR spectra were recorded on a Varian Unity Plus 200 MHz spectrometer operating at 200 MHz for ^1^H and 50 MHz for ^13^C, the sample being dissolved in DMSO-d_6_ with TMS as reference. Elemental analysis was carried out using a Perkin Elmer Elemental Microanalyser. The melting points were determined using a Kofler hot-plate apparatus combined with a Carl-Zeiss microscope and are uncorrected.

*5-Nitro-2-furoyl chloride* (**1**). To a 50 mL flask (protected from moisture by a calcium chloride trap), containing 5-nitro-2-furoic acid (0.50 g, 3.20 mmol) with two drops of dimethylformamide was added rapidly thionyl chloride (0.5 mL). The mixture was heated to 60 ºC for 3 h and then the excess of thionyl chloride was removed in a rotavapor to afford 5-nitro-2-furoyl chloride (yield: 0.41 g, 73.30%) in the form of a yellowish liquid. Being very reactive the acidic chloride was not purified further and was used immediately in the next step of synthesis.

*1,4-Diphenylthiosemicarbazide* (**2**). To phenylhydrazine (12 g, 0.11 mol) dissolved in anhydrous toluene (50 mL) was added phenylisothiocyanate (15 g, 0.11 mol) previously dissolved anhydrous toluene (30 mL) with mechanical stirring. The reaction was heated to 90 °C and after 10 min the product precipitated from the reaction medium. Agitation was continued for a further 120 min, and the mixture allowed to cool to room temperature and filtered under reduced pressure. The precipitate was washed with anhydrous toluene (30 mL) and then with anhydrous ethyl ether (20 mL) and dried under reduced pressure. We obtained 24.8 g (91.9%) of 1,4-diphenylthiosemicarbazide in the form of white acicular crystals, m.p. 175–176 °C (lit. 175–176 ºC, [22]). ^13^C-NMR 181.0, 148.0, 139.0, 129.0, 127.8, 125.1, 124.9, 119.8, 113.0.

*1,4-Diphenyl-5-(5-nitro-2-furanyl)-1,3,4-triazolium-2-thiol chloride* (**MC-1**). To a solution of 1,4-diphenylthiosemicarbazide (0.57 g, 2.3 mmol) in moist dioxane (10 mL) was added piridine (2.3 mmol). After 3 h 5-nitro-2-furoyl chloride (0.41 g, 2.3 mmol, previously dissolved in 5 mL of anhydrous dioxane) was slowly added. After 2 h stirring the reaction mixture was placed in the freezer for 96 h and a precipitate of orange-yellow crystals was formed, (0.4 g, m.p. 263–264 ºC, 45.0%) which was vacuum filtered, and washed with ethanol (10 mL) and cold ether (5 mL) and then air dried. Anal. Calcd for C_18_H_13_ClN_4_O_3_S: C 54.00, H, 3.25; N, 14.00. Found: C, 53.50, H, 3.20; N, 14.30. IR (KBr): 3150, 3040, 2714, 1572, 1351 cm^−1^. ^1^H-NMR (200 MHz, DMSO-d_6_): δ = 8.10–7.20 (m, 12H aromatic), 4.00 (s,1H, sulphydryl). ^13^C-NMR (50 MHz, DMSO-d_6_): δ = 167.8, 152.7, 146.5, 136.6, 132.3, 130.6, 127.7, 133.8, 125.7, 130.6, 127.7, 141.8, 108.7, 114.5.

*4-Phenyl-5-(5-nitro-2-furanyl)-1,3,4-thiadiazolium-2-phenylamine chloride* (**MC-2**). To a solution of 1,4-diphenylthiosemicarbazide (0.57 g, 2.3 mmol) in anhydrous dioxane (10 mL) was added slowly5-nitro-2-furoyl chloride (0.41 g, 2.3 mmol, previously dissolved in 5 mL of anhydrous dioxane). After the addition of the acyl chloride, there was the formation of a yellowish-red solution without warming or gas formation and after a period of 24 h of shaking in a closed system a precipitate of orange-yellow crystals formed (0.6 g, m.p. 261–262 ºC, 66.7%) which were vacuum filtered, and washed with anhydrous dioxane (10 mL) and cold anhydrous ether (5 mL) and then air dried. Anal. Calcd for C_18_H_13_ClN_4_O_3_S: C 54.00, H, 3.25; N, 14.00. Found: C, 53.55, H, 3.30; N, 14.30. IR (KBr): 3444, 3053, 1537, 1345 cm^−1^. ^1^H-NMR (200 MHz, DMSO-d6): δ = 13.20 (s, 1H, NH), 7.93–6.73 (m, 12H aromatic). ^13^C-NMR (50 MHz, DMSO-d6): δ = 166.0, 160.5, 150.7, 139.3, 138.1, 137.6, 132.5, 130.5, 129.5, 125.7, 124.4, 120.7, 118.5, 106.3.

### 3.2. Biological Activity

The heterocyclic compounds **MC-1** and **MC-2** were screened for their biological activities *in vitro* against three effluxing strains of *Staphylococcus aureus*: 1) SA-1199B, which overexpresses the norA gene encoding the NorA efflux protein (resisting fluoroquinolones and others); 2) RN4220, harboring the pUL5054 plasmid, which carries the gene encoding the MsrA macrolid efflux protein; 3) IS-58, which possesses the TetK tetracycline efflux protein [28,29,30,31]. All strains, kindly provided by Professor Simon Gibbons (University of London), were maintained on blood agar base (Difco) slants and, prior to use (assay), the cells were grown overnight at 37 °C in brain heart infusion broth (BHI, Difco).

Norfloxacin, erythromycin and tetracycline were obtained from Sigma Chemical Co., USA. The stock solutions of the antibiotics were prepared according to CLSI guidelines [32]. The stock solution of **MC-1** and **MC-2** was prepared in DMSO, and its highest concentration remaining after dilution into broth (4%) caused no inhibition of bacterial growth.

The minimum inhibitory concentrations (MICs) of the antibiotics and mesoionic compounds were determined in BHI by the microdilution assay using a suspension of ca. 105 cfu/mL and a drug concentration range of 256–0.5 μg/mL (two-fold serial dilutions). For better visualization of bacterial growth (after 24 h) we used resazurin (0.01%). MIC is defined as the lowest concentration at which no growth is observed. For the evaluation of mesoionics as modulators of drug resistance, “modulation assay” was used, a method that has been widely applied to identify potential EPIs, and is valid provided one uses specifically known effluxing strains. The MICs of the antibiotics were determined in the presence of the mesoionic compound at a sub-inhibitory concentration [33,34,35].

## 4. Conclusions

In this study we describe the synthesis and characterization of the novel structure 4-phenyl-5-(5-nitro-2-furanyl)-1,3,4-thiadiazolium-2-phenylamine hydrochloride (**MC-2**). Salts of the mesoionic compounds **MC-1** and **MC-2** show significant biological action, both alone and as adjuvants to common antibiotics, notably affecting *Staphylococcus aureus* resistance to tetracycline, erythromycin. Considering the known singular nature of bacterial resistance to these antibiotics, the results open new perspectives for both **MC-1** and **MC-2** as potential antibiotic adjuvants, and suggest these compounds putatively act as bacterial efflux pump inhibitors. These results also demand further investigation to certify whether these mesoionic derivatives act exclusively through efflux pump inhibition to leverage standard antibiotics against therapy-resistant *Staphylococcus aureus*.
